# Epicardial Adipose Tissue, Functional Status, and Invasive Hemodynamics in Heart Failure With Preserved Ejection Fraction

**DOI:** 10.1016/j.jacadv.2025.102185

**Published:** 2025-08-27

**Authors:** Yoran Crum, Dirk J. van Veldhuisen, Sanjiv J. Shah, Uma Mylavarapu, Mo Hu, Jan Komtebedde, Michelle Lobeek, Elke S. Hoendermis, Sheldon E. Litwin, Scott D. Solomon, Donald E. Cutlip, Martin B. Leon, David Kaye, Leonhard Bruch, Tim Seidler, Maja Cikes, Rajeev Mohan, Scott Lilly, Nicholas Collins, Sitaramesh Emani, Stanley Chetcuti, Heiko Bugger, Eugene Kotlyar, Grant W. Reed, Robert Gordon, Deepak K. Gupta, Barry A. Borlaug, Thomas M. Gorter

**Affiliations:** a University of Groningen, University Medical Centre Groningen, Department of Cardiology, Groningen, The Netherlands; b Northwestern University Feinberg School of Medicine, Chicago, Illinois, USA; c Corvia Medical Inc, Tewksbury, Massachusetts, USA; d Medical University of South Carolina and Ralph H Johnson Veterans Affairs Medical Center, Charleston, South Carolina, USA; e Brigham and Women's Hospital, Boston, Massachusetts, USA; f Beth Israel Deaconess Medical Center, Baim Institute, Boston, Massachusetts, USA; g Columbia University Irving Medical Center/NewYork Presbyterian Hospital, New York, New York, USA; h Cardiovascular Research Foundation, New York, New York, USA; i Faculty of Medicine Nursing and Health Sciences, Monash University, Melbourne, Australia; j BG Klinikum Unfallkrankenhaus Berlin, Berlin, Germany; k Department Cardiology, Kerckhoff-Clinic, Campus of the Justus-Liebig University Gießen, Bad Nauheim, Germany; l Department for Cardiovascular Diseases, University Hospital Centre Zagreb, University of Zagreb School of Medicine, Zagreb, Croatia; m Advanced Heart Failure and Mechanical Circulatory Support Program, Pulmonary Hypertension Program, Division of Cardiology, La Jolla, California, USA; n Department of Cardiology, Ohio State University Wexner Medical Center, Columbus, Ohio, USA; o Department of Cardiology, John Hunter Hospital, Newcastle, New South Wales, Australia; p Lindner Research Center, The Christ Hospital, Cincinnati, Ohio, USA; q Cardiovascular Center, University of Michigan, Ann Arbor, Michigan, USA; r University Heart Center Graz, Department of Cardiology, Medical University of Graz, Graz, Austria, Austria; s Royal Talbot Rehabilitation Centre, Melbourne, Australia; t Department of Cardiovascular Medicine, Cleveland Clinic, Ohio, USA; u Vanderbilt Translational and Clinical Cardiovascular Research Center, Vanderbilt University School of Medicine, Nashville, Tennessee, USA; v Division of Cardiovascular Medicine, Vanderbilt University School of Medicine, Nashville, Tennessee, USA; w Vanderbilt Translational and Clinical Cardiovascular Research Center, Division of Cardiovascular Medicine, Vanderbilt University Medical Center, Nashville, Tennessee, USA; x Department of Cardiovascular Medicine, Mayo Clinic, Rochester, Minnesota, USA

**Keywords:** epicardial adipose tissue, exercise hemodynamics, heart failure with preserved ejection fraction, pathophysiology

## Abstract

**Background:**

Epicardial adipose tissue (EAT) may play a role in the pathophysiology of heart failure (HF) with preserved ejection fraction (HFpEF).

**Objectives:**

This study examined associations between increased EAT, functional status, and invasive exercise hemodynamics in a large cohort of HFpEF patients.

**Methods:**

All patients underwent echocardiography, 6-minute walk distance (MWD) test, Kansas City Cardiomyopathy Questionnaire, and invasive hemodynamic assessment at rest and during ergometry. EAT thickness was measured alongside the right ventricle on echocardiography, expressed in mm and patients were divided according to EAT tertiles.

**Results:**

In total, 566 patients were examined with mean age 72 ± 8 years, 62% women, mean EAT thickness was 6.0 ± 2.4 mm and 11.5% had EAT ≥9 mm. With increasing EAT thickness tertiles, 6-MWD and Kansas City Cardiomyopathy Questionnaire overall summary score were significantly lower (320 [247-385] vs 315 [244-383] vs 287 [210-364] meters, *P* = 0.001; 51 [32-67] vs 45 [32-63] vs 41, [26-56], *P* = 0.003; respectively), whereas the latter was independent of body mass index (*P* = 0.004). At rest, invasive hemodynamics were not different across EAT tertiles. At peak exercise, patients in the highest EAT thickness tertile had higher pulmonary capillary wedge pressure and pulmonary capillary wedge pressure to right atrial pressure gradient, compared to patients in the first and second EAT thickness tertiles (36 ± 8 vs 34 ± 8 mm Hg, *P* = 0.009; 18 ± 7 vs 16 ± 7 mm Hg, *P* = 0.002, respectively).

**Conclusions:**

EAT thickness was associated with impaired quality of life, lower 6-MWD, and higher left-sided filling pressures at peak exercise. Excess EAT may therefore play an important role in functional status and exercise hemodynamics in patients with HFpEF.

Obesity is strongly associated with the development of heart failure (HF) with preserved ejection fraction (HFpEF), but the exact mechanisms remain incompletely defined.^1^, ^2^, ^3^ Although obesity is classically defined as an increase in body mass index (BMI), emerging evidence shows that an abundance of adipose tissue may have deleterious effects on the heart.^4^, ^5^, ^6^, ^7^ In particular, epicardial adipose tissue (EAT) has been suggested to be mechanistically involved in HFpEF, due to its direct proximity to the myocardium.^8^^,^^9^

For instance, EAT may secrete proinflammatory adipokines that may infiltrate the underlying myocardium, disrupt the myocardial structure, and increase myocardial stiffness, leading to diastolic dysfunction and ultimately clinical HFpEF.^9^^,^^10^ In addition, an abundance of EAT within a poorly pliable pericardial sac may exert direct mechanical compression on the myocardium leading to increased filling pressures.^3^^,^^11^^,^^12^ The increase in external constraint from EAT attenuates increases in natriuretic peptides with high filling pressures, concealing the presence of HFpEF by laboratory testing, while exacerbating volume overload through the loss of natriuretic effects.^13^

In earlier studies, an increased EAT has indeed been linked to higher cardiac filling pressures, hemodynamic indicators of pericardial constraint, reduced exercise capacity, and an increased risk for HF hospitalization and mortality, independent of overall obesity.^11^^,^^12^^,^^14^^,^^15^ However, these studies had a small sample size, only had invasive hemodynamics at rest, or were performed exclusively in HFpEF patients with severe obesity. As obesity has emerged as a specific target for therapy in HFpEF, there is an urgent need to better understand the interplay between obesity and EAT on the one hand, and myocardial dysfunction leading to HFpEF on the other hand.

To better investigate the hemodynamic and clinical consequences of EAT in HFpEF, we performed a subanalysis of the REDUCE LAP-HF II (Reduce Elevated Left Atrial Pressure in Patients with Heart Failure II) trial. This trial evaluated the efficacy and safety of an atrial shunt device (Corvia Atrial Shunt System) in patients with HF and left ventricular (LV) ejection fraction (LVEF) ≥40%.^16^^,^^17^ At baseline, all patients underwent echocardiography, 6-minute walk test (MWD), quality of life questionnaire, and subsequently underwent right heart catheterization, both at rest and during supine ergometry. In the present study, we examined the association between the thickness of EAT measured on echocardiography and functional capacity, quality of life, and invasive resting and exercise hemodynamics.

## Methods

The randomized, prospective, double-blinded, sham-controlled REDUCE LAP-HF II trial was conducted at 89 international centers. The study protocol has been previously described.^18^ In brief, 1,072 patients underwent echocardiographic and hemodynamic screening. In total, 626 patients were randomized to atrial shunt or sham procedure. The overall trial result was neutral concerning the primary hierarchical endpoint.^17^

The REDUCE LAP-HF II trial had the following inclusion criteria: 1) age ≥40 years; 2) NYHA functional class II or III, with LVEF ≥40%; 3) elevated left-sided filling pressures defined as end-expiratory pulmonary capillary wedge pressure (PCWP) during supine ergometer exercise ≥25 mm Hg, with PCWP exceeding right atrial pressure (RAP) by ≥5 mm Hg; and 4) structural and/or functional abnormalities indicative of LV diastolic dysfunction on echocardiography (ie, left atrial volume index >28 mL/m^2^, septal e′ <8 cm/s, lateral e′ <10 cm/s, lateral E/e′ >10, and septal E/e′ >15). The major exclusion criteria were as follows: 1) myocardial infarction, percutaneous cardiac intervention, coronary artery bypass grafting, transient ischemic attack, deep vein thrombosis, or pulmonary embolism in the past 6 months; 2) advanced HF (NYHA functional class IV and/or cardiac index <2.0 L/min); and 3) known obstructive/restrictive/infiltrative cardiomyopathy.^18^

The present study is a cross-sectional study, where we used right heart catheterization and transthoracic echocardiographic parameters obtained at baseline. Patients with inadequate echocardiographic quality for assessment of EAT were excluded. The study was approved by the Medical ethical committee of each participating site. All patients provided written informed consent. The study was conducted in concordance with the principles outlined in the Declaration of Helsinki.

### Right heart catheterization protocol

All patients underwent invasive hemodynamic exercise testing as previously described.^17^^,^^19^ In brief, catheterization of the right heart and pulmonary artery was performed via the right internal jugular vein with fluoroscopic guidance. Pressures were assessed using a fluid-filled catheter connected to a properly zeroed pressure transducer. At first, hemodynamic measurements were obtained at rest and subsequently during supine ergometry, which increased by 20 W every 3 minutes until exhaustion. RAP, PCWP, and pulmonary artery pressure were obtained at end-expiration during normal respiration. All hemodynamic measurements were measured at a core laboratory (Cardiovascular Clinical studies, Boston, Massachusetts, USA) and were measured using the average of at least 3 beats. Cardiac output was calculated using the thermodilution method and indexed for body surface area. Pulmonary vascular resistance was calculated using the following equation: (pulmonary vascular resistance = [mean pulmonary artery pressure – PCWP]/cardiac output) and expressed in dynes/s/cm^-5^. The RAP/PCWP ratio was calculated to estimate the degree of pericardial constraint. The PCWP-RAP gradient was subsequently calculated to estimate the transmural pressure.

### Echocardiographic procedure

Echocardiographic measurements were conducted according to the American Society of Echocardiography guidelines.^20^ The echocardiograms were deidentified and subsequently transmitted to the echo core lab (University of Pennsylvania, Philadelphia, Pennsylvania, USA). For the additional EAT measurements, the echocardiographic images were digitally stored and transferred to the University Medical Center Groningen Echo Core Lab using EchoPAC (Version B12, GE HealthCare, Norway).^21^

EAT, which is predominantly located at the lateral free wall of the right ventricle, is defined as the echolucent space between the outer wall of the myocardium and the visceral layer of the pericardium. Based on the current standard, the thickness of EAT was assessed using the parasternal long-axis (with the aortic annulus as an anatomical reference) and parasternal short-axis views (with the papillary muscles and septum as anatomical reference points), both at the end of systole (Central Illustration A).^21^^,^^22^ EAT thickness was measured over 2 cardiac cycles for both the parasternal long- and short-axes views and the 4 measurements were averaged. The EAT thickness was expressed in millimeters and patients were divided according to EAT tertiles. In addition, “excess EAT” was defined as EAT thickness ≥9 mm, based on previous experience.^12^^,^^23^

**Central Illustration:**
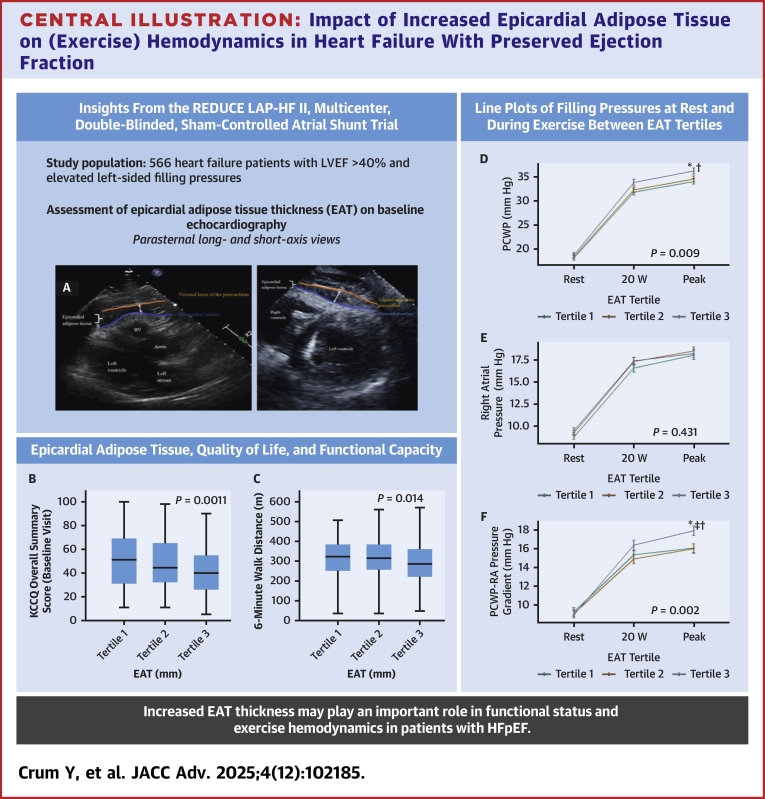
**Impact of Increased Epicardial Adipose Tissue on (Exercise) Hemodynamics in Heart Failure With Preserved Ejection Fraction** (A) Shows an example of EAT assessment at the parasternal long-axis and short-axis views. Using respective the aortic annulus and interventricular septum as reference points. The white arrow indicates the EAT thickness, the blue line the myocardial surface, and orange line the visceral pericardium. (B and C) Shows that increased EAT thickness is associated with lower KCCQ overall summary score and 6-minute walking distance. (D, E, and F) Shows line-plots with significant changes in PCWP and PCWP-RA pressure gradient during peak exercise between EAT tertiles. Boxplots with SE bars displayed for B: KCCQ overall score and C: 6-minute walking distance. *P* value represents trend. ∗*P* < 0.05 between subgroups. §*P* < 0.05 tertile 1 vs tertile 2. †*P* < 0.05 tertile 1 vs tertile 3. ‡*P* < 0.05 tertile 2 vs tertile 3. EAT = epicardial adipose tissue; HFpEF = heart failure with preserved ejection fraction; KCCQ = Kansas City Cardiomyopathy Questionnaire; LVEF = left ventricular ejection fracyion; PCWP = pulmonary capillary wedge pressure; RA = right atrial; REDUCE LAP-HF II = Reduce Elevated Left Atrial Pressure in Patients with Heart Failure II; RV = right ventricle; W = Watt.

### Statistical analyses

Data were presented as mean ± SD or median (IQR) for continuous variables (according to distribution) and as n (%) for categorical measurements. Differences between 2 groups in continuous variables were tested using the independent samples t-test or Mann-Whitney *U* test, as appropriate to distribution. Between-group differences in categorical variables were tested using Fisher exact or chi-square tests. Correlations between continuous variables were tested using Pearson correlation analysis. The thickness of EAT was divided into tertiles and continuous variables among the tertiles were compared using the one-way analysis of variance test or Kruskal-Wallis test according to distribution. We performed univariate and multivariable regression analyses to assess independent associations between EAT thickness, functional status, and hemodynamic parameters. For the multivariable regression model, BMI and sex were included as potential confounders, based on recent literature and our own findings.^3^^,^^19^ The assumption of linearity between the continuous independent variables and dependent variables was assessed using partial regression plots and examination of standardized residuals vs standardized predicted values. No substantial deviations from linearity were observed. For all statistical analyses, 2-tailed *P* < 0.05 and an interaction of *P* < 0.10 were considered statistically significant.

## Results

The REDUCE LAP-HF II trial enrolled a total of 626 patients who underwent a complete assessment at baseline, including echocardiography and invasive exercise right heart catheterization. In total, 566 patients were included in the present study. In 32 patients, the echocardiographic studies could not be technically transferred to our core laboratory. The remaining 28 echocardiograms were of insufficient image quality for the analysis of EAT thickness. Baseline characteristics of the study population and according to the EAT tertiles are depicted in Table 1. The majority were women (n = 339; 61.6%) and the proportion of women significantly increased with increasing EAT tertile (*P* = 0.015). Mean EAT thickness was 6.0 ± 2.4 mm, ranging from 1.7 mm to 17.2 mm, and 65 (11.5%) patients had excess EAT with a thickness ≥9 mm.

**Table 1:** Baseline Characteristics

	Total (N = 566)	1st Tertile (2-5 mm) (n = 187)	2nd Tertile (5-7 mm) (n = 187)	3rd Tertile (7-17 mm) (n = 192)	*P* Value for Trend
Age (y)	72.0 (66.0-77.0)	71.0 (65.0-76.0)	73.0 (66.0-77.0)	72.0 (67.0-77.0)	0.46
Female	339 (61.6%)	103 (56.9%)	107 (57.8%)	129 (70.1%)	**0.015**
Height (cm)	167.3 (160.0-175.0)	167.6 (160.0-174.2)	168.0 (162.0-177.6)	165.0 (159.9-172.3)	**0.011**
Weight (kg)	90.0 (77.0-105.0)	86.0 (73.8-103.0)	90.7 (80.0-106.0)	93.8 (78.8-107.2)	**0.005**
Body mass index (kg/m^2^)	32.1 (27.7-37.0)	30.8 (27.0-35.4)	32.1 (28.0-36.7)	34.2 (29.2-38.1)	**<0.001**
Body surface area (m^2^)	2.0 (1.9-2.2)	2.0 (1.8-2.2)	2.0 (1.9-2.2)	2.1 (1.9-2.3)	**0.016**
Comorbidities					
Hypertension	482 (88.0%)	159 (87.8%)	164 (88.6%)	159 (87.4%)	0.93
Diabetes	210 (38.2%)	61 (33.7%)	68 (36.8%)	81 (44.0%)	0.11
Hyperlipidemia	380 (69.6%)	124 (69.7%)	122 (66.3%)	134 (72.8%)	0.40
Chronic kidney disease	307 (57.3%)	91 (51.1%)	108 (60.7%)	108 (60.0%)	0.13
Chronic obstructive pulmonary disease	113 (20.6%)	37 (20.4%)	34 (18.5%)	42 (22.8%)	0.59
Prior percutaneous coronary intervention	146 (26.7%)	52 (28.7%)	49 (26.8%)	45 (24.7%)	0.69
Coronary artery bypass grafting	52 (9.5%)	20 (11.0%)	14 (7.7%)	18 (9.9%)	0.53
Atrial fibrillation	281 (51.1%)	94 (51.9%)	92 (49.7%)	95 (51.6%)	0.90
Atrial flutter	60 (11.0%)	24 (13.4%)	16 (8.7%)	20 (10.9%)	0.37
Medication					
Loop diuretic agents	459 (83.5%)	155 (85.6%)	151 (81.6%)	153 (83.2%)	0.58
ACE-inhibitors	136 (24.7%)	45 (24.9%)	43 (23.2%)	48 (26.1%)	0.82
Angiotensin-receptor blockers	208 (37.8%)	66 (36.5%)	71 (38.4%)	71 (38.6%)	0.90
Beta-blockers	382 (69.5%)	129 (71.3%)	124 (67.0%)	129 (70.1%)	0.66
MR-antagonists	281 (51.1%)	98 (54.1%)	91 (49.2%)	92 (50.0%)	0.60
SGLT2-inhibitors	16 (2.9%)	1 (0.6%)	7 (3.8%)	8 (4.3%)	0.067

Values are median (Q1-Q3) or n (%). Values in **bold** indicate statistical significance (*P* < 0.05).

ACE = angiotensin converting enzyme; MR = mineralocorticoid receptor; SGLT2 = sodium glucose cotransporter 2.

### Health status and functional capacity

Mean BMI was 33 ± 6 kg/m^2^, and BMI ranged from 18 to 69 kg/m^2^. Across the EAT groups, both weight and BMI were significantly higher with increasing EAT thickness, as seen in Table 1. The medical assessment, symptoms, overall health status, and functional capacity according to EAT tertiles are presented in Table 2. Although NYHA class was comparable among the groups, the Kansas City Cardiomyopathy Questionnaire (KCCQ) clinical summary and overall summary score and the 6-MWD, were significantly lower when the EAT thickness increased (*P* < 0.001, *P* = 0.001, and *P* = 0.014, respectively), as seen in Central Illustration panel B and C and Table 2. In addition, the delta PCWP-RAP gradient was significantly higher in patients with high EAT thickness (*P* < 0.001) (Supplemental Table 1). In the multivariable linear regression model, increased EAT remained significantly associated with the lower KCCQ overall summary score and delta PCWP-RAP gradient after adjustment for BMI and sex, as seen in Supplemental Table 1 (*P* = 0.013; β: −2.3; 95% CI: −4.0 to −0.05 and *P* = 0.009; β: 0.956; 95% CI: 0.210-1.456). The association between increased EAT and lower 6-MWD was not independent of BMI.

**Table 2:** Clinical Assessment, Symptoms, Health Status, and Functional Capacity

	Total (N = 566)	1st Tertile (2-5 mm) (n = 187)	2nd Tertile (5-7 mm) (n = 187)	3rd Tertile (7-17 mm) (n = 192)	*P* Value for Trend
Heart rate (beats/min)	70.0 (63.0-80.0)	70.0 (62.0-80.0)	70.0 (62.0-77.0)	70.0 (63.0-80.0)	0.75
Systolic blood pressure (mm Hg)	143.0 (129.0-158.0)	140.0 (125.0-159.0)	142.0 (130.0-156.0)	145.0 (130.0-160.0)	0.38
Diastolic blood pressure (mm Hg)	74.0 (67.0-82.0)	74.0 (67.0-82.0)	75.0 (68.0-83.0)	74.0 (68.0-83.0)	0.37
NYHA functional class					0.79
I	25 (5.1%)	11 (6.9%)	9 (5.6%)	5 (3.0%)	
II	242 (49.6%)	77 (48.4%)	78 (48.4%)	87 (51.8%)	
III	211 (43.2%)	67 (42.1%)	71 (44.1%)	73 (43.5%)	
IV	10 (2.0%)	4 (2.5%)	3 (1.9%)	3 (1.8%)	
MAGGIC risk score	22.4 ± 5.6	21.8 ± 5.9	22.8 ± 5.5	22.5 ± 5.5	0.29
KCCQ clinical summary score at baseline	50.0 (35.4-67.2)	56.2 (38.0-71.9)	50.0 (36.5-67.7)	44.7 (31.8-59.0)	**<0.001**
KCCQ overall summary score	45.8 (29.2-63.0)	50.5 (31.5-66.9)	45.3 (32.0-63.0)	41.4 (25.9-56.3)	**0.001**
6-minute walk distance (m)	301.4 (235.0-380.0)	320.0 (246.9-385.0)	315.0 (244.1-382.8)	287.0 (210.2-363.9)	**0.014**
Ejection fraction (%)	60.0 (55.0-65.0)	60.0 (55.0-65.0)	60.0 (55.0-65.0)	60.0 (55.0-66.0)	0.24
HF subtype (Baim database)					0.27
HFmrEF (EF 41-49%)	85 (17.3%)	32 (20.3%)	30 (18.3%)	23 (13.7%)	
HFpEF (EF ≥ 50%)	405 (82.7%)	126 (79.7%)	134 (81.7%)	145 (86.3%)	
H2FPEF score	6.0 (5.0-8.0)	6.0 (4.0-7.0)	6.0 (5.0-8.0)	6.0 (5.0-8.0)	0.48
At least 1 hospitalization for HF in the past 12 months	155 (28.2%)	47 (26.0%)	49 (26.5%)	59 (32.1%)	0.35

Values are median (Q1-Q3), mean ± SD, or n (%). Values in **bold** indicate statistical significance (*P* < 0.05).

EF = ejection fraction; H2FPEF = heart 2 failure with preserved ejection fraction; HF = heart failure; HFmrEF = heart failure with mildly reduced ejection fraction; HFpEF = heart failure with preserved ejection fraction; KCCQ = Kansas City Cardiomyopathy Questionnaire; MAGGIC = Meta-Analysis Global Group in Chronic Heart Failure.

### Echocardiographic parameters

Most echocardiographic parameters were comparable between EAT tertiles. However, LV end-diastolic volume index, right ventricular end-diastolic diameter, and right atrial volume were significantly lower when EAT thickness increased (*P* = 0.022, *P* = 0.016, and *P* = 0.038, respectively) (Supplemental Table 2). Mitral valve peak E-wave velocity was lower and mitral valve peak A-wave velocity was higher in patients with the highest EAT thickness, but E/e′ ratio was not different across EAT tertiles. Left atrial volume and left atrial reservoir strain were not significantly different across EAT tertiles.

### Invasive hemodynamics at rest and during exercise

At rest, most of the invasive hemodynamic parameters were comparable among the EAT tertiles. In particular, RAP, PCWP, and the PCWP-RAP gradient were not different across EAT tertiles, as seen in Central Illustration panel D to F and Table 3.

**Table 3:** Hemodynamic Parameters at Rest and During Exercise

	Total (N = 566)	1st Tertile (2-5 mm) (n = 187)	2nd Tertile (5-7 mm) (n = 187)	3rd Tertile (7-17 mm) (n = 192)	*P* Value for Trend
Rest					
Heart rate (beats/min)	70.0 (63.0-80.0)	70.0 (62.0-80.0)	70.0 (62.0-77.0)	70.0 (63.0-80.0)	0.75
Systolic blood pressure (mm Hg)	144.0 (129.0-158.0)	141.5 (125.0-160.0)	142.0 (130.0-156.0)	145.5 (131.5-160.5)	0.46
Diastolic blood pressure (mm Hg)	75.0 (68.0-83.0)	74.0 (67.0-82.0)	75.0 (68.0-83.0)	74.0 (68.0-83.0)	0.67
Right atrial pressure (mm Hg)	9.0 (6.0-12.0)	9.0 (6.0-11.0)	9.0 (7.0-11.0)	10.0 (7.0-12.0)	0.18
PA systolic pressure (mm Hg)	40.0 (33.0-49.0)	40.0 (33.0-50.0)	39.0 (34.0-49.0)	40.0 (33.0-50.0)	0.88
PA diastolic pressure (mm Hg)	19.0 (15.0-23.0)	18.0 (15.0-22.0)	19.0 (15.0-24.0)	20.0 (16.0-25.0)	0.052
Mean PA pressure (mm Hg)	26.0 (21.3-32.0)	25.3 (21.3-31.0)	25.7 (20.7-32.3)	26.7 (22.0-33.0)	0.31
PCWP (mm Hg)	17.0 (14.0-23.0)	18.0 (12.0-23.0)	17.0 (14.0-22.0)	17.0 (14.0-23.0)	0.77
PCWP-RA pressure gradient (mm Hg)	8.0 (5.0-12.0)	8.0 (5.0-12.0)	9.0 (5.0-12.0)	8.0 (5.0-12.0)	0.95
RAP/PCWP ratio	0.5 (0.4-0.6)	0.5 (0.4-0.6)	0.5 (0.4-0.6)	0.5 (0.4-0.6)	0.59
Transpulmonary gradient (mm Hg)	8.0 (6.0-11.0)	8.0 (6.0-10.0)	8.2 (5.7-11.0)	8.7 (6.3-11.7)	0.25
Stroke volume (mL)	75.3 (63.1-89.6)	74.6 (61.2-88.0)	77.4 (64.0-90.2)	74.4 (63.9-89.8)	0.39
Stroke volume index (mL/m^2^)	36.9 (31.7-43.8)	37.4 (31.6-43.9)	36.8 (31.7-43.9)	36.8 (32.1-42.8)	0.98
Cardiac output, L/min (Baim adjudicated)	5.2 (4.4-6.2)	5.0 (4.3-6.0)	5.4 (4.5-6.3)	5.3 (4.5-6.3)	0.19
Cardiac index (L/min/m^2^)	2.6 (2.2-3.0)	2.5 (2.2-3.0)	2.6 (2.2-3.1)	2.6 (2.2-3.0)	0.99
Systemic vascular resistance (WU)	16.8 (13.7-20.9)	17.6 (13.9-21.7)	16.2 (13.8-19.6)	16.6 (13.7-21.6)	0.17
PVR, WU (Baim adjudicated)	1.5 (1.1-2.1)	1.5 (1.1-2.1)	1.5 (1.0-2.0)	1.6 (1.2-2.2)	0.30
PA pulse pressure/stroke volume ratio (mm Hg/mL)	0.3 (0.2-0.4)	0.3 (0.2-0.4)	0.3 (0.2-0.3)	0.3 (0.2-0.4)	0.28
Peak exercise					
Peak workload	40.0 (20.0-60.0)	40.0 (20.0-60.0)	40.0 (20.0-60.0)	40.0 (20.0-60.0)	0.34
Total duration of exercise (minutes)	8.0 (5.0-11.0)	8.0 (5.0-11.0)	7.5 (6.0-11.0)	7.0 (5.0-11.0)	0.81
Peak heart rate (beats/min)	100.5 (86.0-114.0)	102.0 (89.0-115.0)	100.0 (85.0-114.0)	99.0 (86.0-112.5)	0.32
Systolic blood pressure (mm Hg)	161.8 ± 31.1	161.3 ± 33.4	161.0 ± 29.6	163.1 ± 30.4	0.81
Diastolic blood pressure (mm Hg)	85.0 (72.0-96.0)	85.0 (70.0-94.0)	86.0 (72.0-96.0)	85.0 (75.0-96.0)	0.62
Right atrial pressure (mm Hg)	18.0 (14.0-22.0)	18.0 (14.0-22.0)	18.0 (14.0-22.0)	18.0 (14.0-22.0)	0.61
PA systolic pressure (mm Hg)	161.8 (31.1)	161.3 (33.4)	161.0 (29.6)	163.1 (30.4)	0.81
PA diastolic pressure (mm Hg)	34.0 (29.0-40.0)	33.0 (28.0-40.0)	32.0 (29.0-39.0)	35.0 (30.0-40.0)	**0.040**
Mean PA pressure (mm Hg)	45.3 (39.7-52.0)	44.7 (39.3-52.0)	44.0 (39.7-51.3)	46.0 (40.7-52.3)	0.41
PCWP (mm Hg)	34.0 (29.0-40.0)	33.0 (28.0-40.0)	33.0 (29.0-39.0)	35.0 (30.5-41.0)	0.056
PCWP-RA pressure gradient (mm Hg)	16.0 (12.0-21.0)	16.0 (12.0-20.0)	16.0 (11.5-19.0)	18.0 (13.0-22.0)	**0.029**
RAP/PCWP ratio	0.5 (0.4-0.6)	0.5 (0.4-0.6)	0.5 (0.4-0.6)	0.5 (0.4-0.6)	0.22
Transpulmonary gradient (mm Hg)	10.7 (7.3-15.0)	10.7 (7.3-16.0)	10.8 (7.3-14.7)	10.7 (7.3-15.0)	0.94
Stroke volume (mL)	53.0 (43.5-66.5)	50.0 (41.3-63.8)	56.0 (45.1-69.2)	53.6 (44.7-64.3)	0.065
Cardiac output, L/min (Baim adjudicated)	8.1 (6.4-10.2)	7.9 (6.4-10.2)	8.2 (6.5-10.3)	8.2 (6.4-10.1)	0.49
Systemic vascular resistance (WU)	11.4 (8.8-14.7)	12.2 (8.8-15.5)	11.2 (8.7-14.2)	11.2 (8.9-14.6)	0.42
PVR, WU (Baim adjudicated)	1.3 (0.8-2.0)	1.3 (0.9-2.0)	1.3 (0.8-1.9)	1.4 (0.8-2.0)	0.62
PA pulse pressure/stroke volume ratio (mm Hg/mL)	0.6 (0.5-0.9)	0.7 (0.5-1.0)	0.6 (0.5-0.8)	0.6 (0.5-0.8)	**0.028**
Workload-corrected PCWP (mm Hg/W/kg)	75.7 (50.5-122.4)	73.0 (44.3-105.8)	69.3 (51.3-123.3)	82.1 (55.8-145.3)	**0.017**
PCWP/CO slope (mm Hg/L/min)	5.7 (3.5-10.0)	5.5 (3.4-10.2)	5.2 (3.4-9.2)	6.1 (3.8-10.3)	0.31

Values are median (Q1-Q3) or mean ± SD. The Shapiro-Wilk test was used to determine normality of each variable. Values in **bold** indicate statistical significance (*P* < 0.05).

CO = cardiac output; PA = pulmonary artery; PCWP = pulmonary capillary wedge pressure; PVR, pulmonary vascular resistance; RA = right atrial; RAP = right atrial pressure; WU = Wood unit.

During exercise, several significant differences were revealed in invasive hemodynamics between EAT tertiles. At 20 W exercise, cardiac filling pressures such as RAP, PCWP, and the PCWP-RAP gradient increased in the entire population, with a steeper increase in PCWP and the PCWP-RAP gradient in the highest tertile, as seen in Central Illustration D to F and Supplemental Table 3. Especially at peak exercise, significant differences were present in invasive hemodynamics between the EAT groups (Central Illustration D to F, Table 3). At peak exercise, there was a higher increase in PCWP in the group with the highest EAT tertile, but the increase in RAP at peak exercise was comparable between the EAT groups (Central Illustration D and E, Supplemental Tables 3 and 4). Hence, there was a pronounced difference in the increase in PCWP and the PCWP-RAP gradient between the EAT tertiles, with patients in the highest EAT tertile having a significantly higher PCWP (36 ± 8 vs 34 ± 8 mm Hg; *P* = 0.009) and PCWP-RAP gradient (18 ± 7 vs 16 ± 7 mm Hg; *P* = 0.002) at peak exercise, as compared to patients in the other EAT tertiles (Central Illustration D and F, Supplemental Table 4). In contrast, the RAP/PCWP ratio remained comparable between all EAT tertiles at all exercise stages. Also when patients were divided into 2 groups according to EAT <9 and ≥ 9 mm, the PCWP-RAP gradient at peak exercise was higher for patients with EAT ≥9 mm as compared to those with EAT <9 mm (19 ± 6 vs 16 ± 7 mm Hg, *P* = 0.020, respectively), as well as the change in PCWP-RAP gradient from rest to peak exercise (ie, delta +10 ± 7 vs +7 ± 7 mm Hg, *P* = 0.010, respectively).

## Discussion

The present study is the largest to investigate the associations between EAT and invasive exercise hemodynamics. The results show that a large proportion of patients with HF and LVEF ≥40% have a significant amount of EAT alongside the right ventricular free wall, with maximal EAT thickness exceeding 17 mm and 65 patients (12%) having excess EAT thickness of ≥9 mm. Furthermore, we observed an important association between increased EAT thickness and higher left-sided filling pressures. Interestingly, however, the associations between higher EAT thickness and increased left-sided filling pressures were not present at rest and only became evident during exercise. Indeed, patients with high EAT thickness had a larger increase in PCWP and PCWP-RAP gradient at peak exercise, as compared to patients with lower EAT thickness. In addition, an increase in EAT thickness was associated with lower 6-MWD and worse KCCQ scores, with the association between EAT and low KCCQ score being independent of BMI. This study thus indicates that EAT may play an important role in functional status and exercise hemodynamics in patients with HFpEF.

In the present study, we observed that during exercise, PCWP and the PCWP-RAP gradient increased to a higher extent in patients with higher EAT thickness as compared to those with less EAT thickness, as no difference in RAP was seen at peak exercise between the groups. In contrast to our study, Koepp et al have reported that increased EAT was predominantly associated with higher RAP during exercise, suggesting more pericardial restraint.^12^ A possible explanation for this discrepancy is the selected population consisting exclusively of patients with severe obesity with mean BMI 37 kg/m^2^ and on average much higher EAT thickness, namely ∼8.5 mm with 46% of their population having excess EAT thickness ≥9 mm. In the present study, mean BMI was considerably lower, with an average EAT thickness of 6 mm. Interestingly, however, in a recent subanalysis from the same REDUCE LAP-HF II population, Litwin et al also revealed that obesity was associated with a higher increase in PCWP and especially in RAP during exercise, with a comparable increase in PCWP-RAP gradient during exercise across subgroups with increasing BMI.^19^ A similar phenomenon was seen in 2 other studies of obesity-related HFpEF.^3^^,^^4^ It may be that a selection based on overall adiposity may result in an over-representation of patients with high circulating plasma volume since dysfunctional visceral adipose tissue in patients with obesity secretes leptin, aldosteron, and neprilysin, which leads to sodium retention.^24^ The resulting plasma volume expansion is directly correlated with an increase in biventricular filling pressures in obesity-related HFpEF.^3^^,^^4^

Our population was not selected according to increased BMI and it is possible that the correlation between increased EAT thickness and increased left-sided filling pressures during exercise may be explained by direct infiltrative-lipotoxic properties of EAT and not by systemic and circulating factors. This infiltration may lead to more interstitial fibrosis and enhanced restrictive properties of the underlying myocardium, resulting in diastolic dysfunction and higher increase in filling pressures during exercise.^8^, ^9^, ^10^

Indeed, we demonstrated that the increased EAT thickness was significantly associated with reduced 6-MWD and KCCQ scores. Furthermore, the association between increased EAT and reduced KCCQ score was independent of overall obesity. Our findings align with another study that also reported that high EAT thickness was associated with lower KCCQ scores. However, in that study population, this association was largely driven by increased BMI.^25^ To the best of our knowledge, ours is the first study to describe this association in such a large study population, while also demonstrating its independence from BMI. In addition, we showed that the association between EAT and KCCQ scores was strongest for the KCCQ clinical summary score, which is more specific to HFpEF and less for overall obesity. A potential explanation may be the observed higher increase PCWP in patients with an abundance of EAT, causing exercise-induced pulmonary venous hypertension with more consistent symptoms. Increase in external cardiac compression has been proposed to drive increase in PCWP, but if that is the cause in patients with high EAT, we would have expected equivalent or greater increases in RAP, as a measure of pericardial pressure.^26^ Instead, the greater increases in PCWP out of proportion to RAP suggest more severe impairment in left heart function that was unmasked with exercise.

### Clinical implications

Although there is an increasing interest in EAT, it is still unclear whether EAT plays a significant, solitary role in the pathophysiology of HFpEF and whether EAT may be a potential therapeutic target. First, emerging evidence now suggests that for the development and clinical course of cardiovascular disease, including HFpEF, one has to look beyond BMI to the actual amount and distribution of visceral adipose tissue.^4^, ^5^, ^6^^,^^15^ We showed that, in a large cohort of patients with HFpEF, who were not selected on overall adiposity, the assessment of EAT thickness provides additional mechanistic insights into the potential causes of increased filling pressures and impaired functional status. Recent studies have highlighted the challenges in the diagnosis of the severity of illness in patients with HFpEF. Although natriuretic peptides are commonly employed as a diagnostic marker, it has been argued that patients with more advanced HFpEF often do not have elevated natriuretic peptide levels.^27^ Indeed, measurements of natriuretic peptides have not substantially contributed to identifying disease severity in this HFpEF population.^28^ In contrast to natriuretic peptides, we and others have now demonstrated that increased EAT may also play a role as a diagnostic marker of unexplained dyspnea and exercise intolerance.

To date, there are no proven treatment options that specifically target EAT. However, certain treatments for obesity and type 2 diabetes mellitus have been found to concurrently reduce EAT. Although the mechanisms underlying these observed effects remain unclear, both sodium-glucose cotransporter 2 inhibitors and glucagon-like peptide 1 agonists may be associated with a reduction in EAT and its proinflammatory characteristics.^29^, ^30^, ^31^ However, sodium-glucose cotransporter-2 inhibitors and glucagon-like peptide-1agonists also induce overall weight loss, lower blood pressure, and improve blood glucose regulation, although sodium-glucose cotransporter-2 inhibitors do appear to have greater effects on trunk and android fat in HFpEF, which is related to EAT.^32^ In addition, in a subanalysis from the study of Tirzepatide in Participants with Heart Failure with Preserved Ejection fraction and Obesity (SUMMIT) trial, the dual glucose-dependent insulinotropic polypeptide and glucagon-like peptide-1 agonist tirzepatide mainly reduced extrapericardial adipose tissue, the adipose tissue situated outside the parietal pericardium, which is less likely to cause pericardial constraint, and not EAT itself.^33^ Therefore, it remains challenging to determine to what extent the beneficial effects of these emerging treatments for HFpEF patients are mediated via a reduction in EAT.

### Study Limitations

This study has several potential limitations. First, this was a trial population with specific inclusion and exclusion criteria that may have led to selection bias and limited generalizability. Second, this was a post hoc analysis and the study was not specifically designed to investigate the associations between EAT and invasive hemodynamics. Third, alternative anthropometric measures, such as waist-to-hip ratio and waist circumference, were not available from the REDUCE LAP-HF II trial. These parameters may have provided additional insight into the importance of fat distribution and invasive hemodynamics in HFpEF. Fourth, although we had invasive hemodynamics during exercise, exercise echocardiography was not performed. Lastly, both cardiac magnetic resonance and computed tomography provide a more precise analysis of overall EAT and informs on the EAT distribution.^21^^,^^34^ However, cardiac magnetic resonance and computed tomography are often not available whereas echocardiography has been demonstrated to be a useful alternative for the initial assessment of EAT.^21^

## Conclusions

This study demonstrates that many patients with HF and LVEF ≥40% have an abundance of EAT. Increased EAT thickness in these patients was associated with lower quality of life, delta PCWP-RAP gradient, and functional capacity, although the latter was not independent of BMI and sex. In the overall population, patients with increased EAT thickness have significantly increased left-sided filling pressures and transmural pressure during exercise. This association was not seen under resting conditions. These data suggest that EAT may play an important role in functional status and exercise hemodynamics in patients with HFpEF.

Perspectives **COMPETENCY IN MEDICAL KNOWLEDGE:** It has been proposed that EAT might be mechanistically involved in the pathophysiology of HFpEF, due to its direct proximity to the myocardium. In the present study, we demonstrated that in patients with HFpEF, increased EAT was associated with lower quality of life and functional capacity. We also showed that patients with excess EAT had significantly higher left-sided filling pressures and transmural pressures. This association was not present at rest but was only revealed during exercise. This might suggest that EAT is not only an innocent bystander, but indeed plays a significant clinical role in HFpEF.

**TRANSLATIONAL OUTLOOK:** The results of the present study provides further insight into the association of EAT with the complex and heterogenous clinical characteristics of HFpEF. Furthermore, as seen in recent studies, challenges have been highlighted in the diagnosis of the severity of illness in HFpEF patients. In contrast to measurements of natriuretic peptides, which have not adequately contributed to identifying disease severity, EAT may play a significant role as a diagnostic marker of unexplained dyspnea and exercise intolerance.

## Funding support and author disclosures

This study was sponsored by Corvia Medical Inc. Dr Veldhuisen reported being a member of the steering committee of the REDUCE-LAP trial but not receiving payments from the sponsor. Dr Shah has received research grants from the 10.13039/100000002National Institutes of Health (U54 HL160273, R01 HL107577, R01 HL127028, R01 HL140731, R01 HL149423), Actelion, 10.13039/100004325AstraZeneca, Corvia, 10.13039/100004336Novartis, and 10.13039/100004319Pfizer; and has received personal fees from Abbott, Actelion, AstraZeneca, Amgen, Aria CV, Axon Therapies, Bayer, Boehringer Ingelheim, Boston Scientific, Bristol Myers Squibb, Cardiora, Coridea, CVRx, Cyclerion, Cytokinetics, Edwards Lifesciences, Eidos, Eisai, Imara, Impulse Dynamics, Intellia Therapeutics, Ionis, Ironwood, Lilly, Merck, MyoKardia, Novartis, Novo Nordisk, Pfizer, Prothena, Regeneron, Rivus, Sanofi, Shifamed, Tenax, Tenaya, and United Therapeutics. Dr Komtebedde is employed by Corvia. Dr Litwin has received research funding from the department of 10.13039/100000738Veterans Affairs, Corvia, 10.13039/100004325AstraZeneca, V-Wave, and Axon Therapeutics. Dr Solomon has received research grants from 10.13039/100006400Alnylam, 10.13039/100004325AstraZeneca, Bellerophon, 10.13039/100004326Bayer, Bristol Myers Squibb, 10.13039/100014941Cytokinetics, Eidos, 10.13039/100004330GlaxoSmithKline, Ionis, 10.13039/100004312Lilly, 10.13039/100016619MyoKardia, the 10.13039/100000002National Institutes of Health/10.13039/100000050National Heart, Lung, and Blood Institute, 10.13039/100004336Novartis, 10.13039/501100004191Novo Nordisk, 10.13039/100019040Respicardia, 10.13039/100014588Sanofi Pasteur, Theracos, and US2.AI; and has consulted for Abbott, Action, Akros, Alnylam, Amgen, Arena, AstraZeneca, Bayer, Boehringer Ingelheim, Bristol Myers Squibb, Cardior, Cardurion, Corvia, Cytokinetics, Daiichi-Sankyo, GlaxoSmithKline, Lilly, Merck, Myokardia, Novartis, Roche, Theracos, Quantum Genomics, Cardurion, Janssen, Cardiac Dimensions, Tenaya, Sanofi-Pasteur, DiNAQOR, Tremeau, CellProthera, Moderna, American Regent, Sarepta, Lexicon, AnaCardio, and Akros. Dr Cutlip has received research support from Corvia paid to the institution; has received research support from Corvia paid to the institution. Dr Kaye has received research support from Corvia. Dr Cikes has received institutional research grants from Abbott, Novartis, and Pfizer; has received travel grants, speaker fees, and advisory board honoraria from Abbott, Abiomed, Amicus, AstraZeneca, Bayer, Boehringer Ingelheim, GE Healthcare, Krka Pharma, LivaNova, Medtronic, Novartis, Orion Corporation, Pfizer, Sanofi, Swixx BioPharma, and Teva Pharmaceutical Industries, all outside of the present study; and has received research support from Corvia. Dr Mohan has received research support from Corvia and V-Wave paid to the institution. Dr Gupta has received research support from the 10.13039/100000002National Institutes of Health, Imara, Corvia, and 10.13039/501100004948Astellas Pharma. Dr Borlaug has received research grants from Corvia, AstraZeneca, Medtronic, GlaxoSmithKline, Mesoblast, Novartis, and Tenax Therapeutics; and has received consulting fees from Actelion, Amgen, Aria, Axon Therapies, Boehringer Ingelheim, Edwards Lifesciences, Eli Lilly, Imbria, Janssen, Merck, Novo Nordisk, and VADovations. Dr Gorter is supported by the Mandema-Stipend of the Junior Scientific Masterclass 2020-10 of the University Medical Centre Groningen and reports nonpersonal consultancy fees from Corvia Medical paid directly to the Institution. All other authors have reported that they have no relationships relevant to the contents of this paper to disclose.

## References

[1] Borlaug B.A., Jensen M.D., Kitzman D.W., Lam C.S.P., Obokata M., Rider O.J. (2023). Obesity and heart failure with preserved ejection fraction: new insights and pathophysiological targets. Cardiovasc Res.

[2] Savji N., Meijers W.C., Bartz T.M. (2018). The association of obesity and cardiometabolic traits with incident HFpEF and HFrEF. JACC Heart Fail.

[3] Obokata M., Reddy Y.N.V., Pislaru S.V., Melenovsky V., Borlaug B.A. (2017). Evidence supporting the existence of a distinct obese phenotype of heart failure with preserved ejection fraction. Circulation.

[4] Sorimachi H., Obokata M., Takahashi N. (2021). Pathophysiologic importance of visceral adipose tissue in women with heart failure and preserved ejection fraction. Eur Heart J.

[5] Peikert A., Vaduganathan M., Claggett B.L. (2025). Near-universal prevalence of central adiposity in heart failure with preserved ejection fraction: the PARAGON-HF trial. Eur Heart J.

[6] Kenchaiah S., Ding J., Carr J.J. (2021). Pericardial fat and the risk of heart failure. J Am Coll Cardiol.

[7] Ramo J.T., Kany S., Hou C.R. (2024). Cardiovascular significance and genetics of epicardial and pericardial adiposity. JAMA Cardiol.

[8] van Woerden G., van Veldhuisen D.J., Westenbrink B.D., de Boer R.A., Rienstra M., Gorter T.M. (2022). Connecting epicardial adipose tissue and heart failure with preserved ejection fraction: mechanisms, management and modern perspectives. Eur J Heart Fail.

[9] Packer M. (2018). Epicardial adipose tissue may mediate deleterious effects of obesity and inflammation on the myocardium. J Am Coll Cardiol.

[10] Wu C.K., Lee J.K., Hsu J.C. (2020). Myocardial adipose deposition and the development of heart failure with preserved ejection fraction. Eur J Heart Fail.

[11] Crum Y., Hoendermis E.S., van Veldhuisen D.J. (2024). Epicardial adipose tissue and pericardial constraint in heart failure with preserved ejection fraction. ESC Heart Fail.

[12] Koepp K.E., Obokata M., Reddy Y.N.V., Olson T.P., Borlaug B.A. (2020). Hemodynamic and functional Impact of epicardial adipose tissue in heart failure with preserved ejection fraction. JACC Heart Fail.

[13] Obokata M., Reddy Y.N.V., Melenovsky V., Sorimachi H., Jarolim P., Borlaug B.A. (2022). Uncoupling between intravascular and distending pressures leads to underestimation of circulatory congestion in obesity. Eur J Heart Fail.

[14] Gorter T.M., van Woerden G., Rienstra M. (2020). Epicardial adipose tissue and invasive hemodynamics in heart failure with preserved ejection fraction. JACC Heart Fail.

[15] van Woerden G., van Veldhuisen D.J., Manintveld O.C. (2022). Epicardial adipose tissue and outcome in heart failure with Mid-range and preserved ejection fraction. Circ Heart Fail.

[16] Patel R.B., Silvestry F.E., Komtebedde J. (2024). Atrial shunt device effects on cardiac structure and function in heart failure with preserved ejection fraction: the REDUCE LAP-HF II randomized clinical trial. JAMA Cardiol.

[17] Shah S.J., Borlaug B.A., Chung E.S. (2022). Atrial shunt device for heart failure with preserved and mildly reduced ejection fraction (REDUCE LAP-HF II): a randomised, multicentre, blinded, sham-controlled trial. Lancet.

[18] Berry N., Mauri L., Feldman T. (2020). Transcatheter InterAtrial shunt device for the treatment of heart failure: rationale and design of the pivotal randomized trial to REDUCE elevated left atrial pressure in patients with heart failure II (REDUCE LAP-HF II). Am Heart J.

[19] Litwin S.E., Komtebedde J., Seidler T. (Jan 2024). Obesity in heart failure with preserved ejection fraction: insights from the REDUCE LAP-HF II trial. Eur J Heart Fail.

[20] Lang R.M., Badano L.P., Mor-Avi V. (2015). Recommendations for cardiac chamber quantification by echocardiography in adults: an update from the American Society of Echocardiography and the European Association of Cardiovascular Imaging. J Am Soc Echocardiogr.

[21] van Woerden G., van Veldhuisen D.J., Gorter T.M. (2022). The value of echocardiographic measurement of epicardial adipose tissue in heart failure patients. ESC Heart Fail.

[22] Iacobellis G. (2022). Epicardial adipose tissue in contemporary cardiology. Nat Rev Cardiol.

[23] Iacobellis G., Willens H.J., Barbaro G., Sharma A.M. (Apr 2008). Threshold values of high-risk echocardiographic epicardial fat thickness. Obesity (Silver Spring).

[24] Packer M. (2018). Leptin-aldosterone-neprilysin Axis: identification of its distinctive role in the pathogenesis of the three phenotypes of heart failure in people with obesity. Circulation.

[25] Venkateshvaran A., Faxen U.L., Hage C. (2022). Association of epicardial adipose tissue with proteomics, coronary flow reserve, cardiac structure and function, and quality of life in heart failure with preserved ejection fraction: insights from the PROMIS-HFpEF study. Eur J Heart Fail.

[26] Borlaug B.A., Reddy Y.N.V. (2019). The role of the pericardium in heart failure: implications for pathophysiology and treatment. JACC Heart Fail.

[27] Packer M. (2011). Can brain natriuretic peptide be used to guide the management of patients with heart failure and a preserved ejection fraction? The wrong way to identify new treatments for a nonexistent disease. Circ Heart Fail.

[28] Verbrugge F.H., Omote K., Reddy Y.N.V., Sorimachi H., Obokata M., Borlaug B.A. (2022). Heart failure with preserved ejection fraction in patients with normal natriuretic peptide levels is associated with increased morbidity and mortality. Eur Heart J.

[29] Margulies K.B., Hernandez A.F., Redfield M.M. (2016). Effects of liraglutide on clinical stability among patients with advanced heart failure and reduced ejection fraction: a randomized clinical trial. JAMA.

[30] Chen W.R., Hu S.Y., Chen Y.D. (2015). Effects of liraglutide on left ventricular function in patients with ST-segment elevation myocardial infarction undergoing primary percutaneous coronary intervention. Am Heart J.

[31] Launbo N., Zobel E.H., von Scholten B.J., Faerch K., Jorgensen P.G., Christensen R.H. (2021). Targeting epicardial adipose tissue with exercise, diet, bariatric surgery or pharmaceutical interventions: a systematic review and meta-analysis. Obes Rev.

[32] Naser J.A., Tada A., Harada T. (2024). Effects of dapagliflozin on body composition and its relation to hemodynamics in heart failure with preserved ejection fraction. Circulation.

[33] Kramer C.M., Borlaug B.A., Zile M.M.R. (2024). Tirzepatide reduces LV mass and paracardiac adipose tissue in obesity-related heart failure: SUMMIT CMR substudy. J Am Coll Cardiol.

[34] Guglielmo M., Lin A., Dey D. (2021). Epicardial fat and coronary artery disease: role of cardiac imaging. Atherosclerosis.

