# Supplementary material for: Epicardial Adipose Tissue, Functional Status, and Invasive Hemodynamics in Heart Failure With Preserved Ejection Fraction
Source: JACC Adv. 2025 Aug 27;4(12):102185. doi: 10.1016/j.jacadv.2025.102185 (PMC12793855; doi:10.1016/j.jacadv.2025.102185)
Supplement: Supplemental Material [file mmc1.pdf]

Supplemental Table 1: Association between EAT, quality of life, 6-minute walking distance and delta transmural pressure.

Outcome	All patients (unadjusted) (n=550)		All patients (adjusted for BMI and sex) (n=550)		BMI≤30 kg/m^2 (n=210)		BMI>30 kg/m^2 (n=340)	
	β (95% CI)*	P-value	β (95% CI)*	P-value	β (95% CI)*	P-value	β (95% CI)*	P-value
KCCQ-OSS	-3.3 (-5.1, -1.5)	<.001	-2.3 (-4.0, -0.5)	.013	-3.6 (-6.7, -0.5)	.023	-2.4 (-4.5, -0.2)	.031
6-MWD	-11.5 (-20.2, -2.7)	.010	-5.6 (-14.2, 3.0)	.20	-6.9 (-21.8, 8.06)	.37	-10.5 (-21.2, 0.2)	.053
Delta PCWP-RAP gradient	1.409 (0.437, 1.662)	<.001	0.956 (0.210, 1.456)	.009	1.023 (0.089, 1.957)	.032	0.990 (0.171, 1.809)	.018

Beta coefficient with 95% confidence interval (CI) for the association between EAT and clinical parameters. *Per 1-SD increase in epicardial adipose tissue BMI, body mass index; EAT, epicardial adipose tissue; KCCQ-OSS, Kansas City Cardiomyopathy Questionnaire Overall Summary Score; 6-MWD, 6-minute walking distance; PCWP, pulmonary capillary wedge pressure; RAP, right atrial pressure.

Supplemental Table 2: Echocardiographic parameters.

	Total N=566	1st tertile (2 – 5mm) N=187	2nd tertile (5 – 7mm) N=187	3rd tertile (7 – 17mm) N=192	p-value
LV end-diastolic dimension (cm)	4.8 (4.4-5.3)	4.8 (4.3-5.2)	4.8 (4.4-5.2)	4.9 (4.5-5.3)	.13
LV end-systolic dimension (cm)	3.5 (3.1-4.0)	3.4 (2.9-3.9)	3.5 (3.2-4.1)	3.5 (3.2-4.0)	.069
LV mass (g)	163.3 (135.8-203.3)	162.1 (131.2-189.3)	158.8 (132.8-206.7)	165.7 (142.5-212.5)	.11
Relative wall thickness	0.4 (0.3-0.4)	0.4 (0.3-0.4)	0.4 (0.3-0.4)	0.4 (0.3-0.4)	.72
LV end-diastolic volume (ml)	109.0 (91.0-136.0)	109.5 (91.0-137.0)	112.0 (92.4-137.2)	105.0 (87.0-130.0)	.17
LV end-systolic volume (ml)	51.0 (40.0-66.1)	52.0 (41.0-68.2)	52.0 (41.8-65.2)	48.0 (39.0-61.0)	.11
LV end-diastolic volume index (ml/m2)	54.0 (46.0-65.0)	57.2 (48.4-66.7)	53.6 (46.4-64.9)	52.3 (43.3-62.8)	**.022**
LV end-systolic volume index (ml/m2)	25.1 (20.4-31.7)	26.5 (21.3-33.4)	25.3 (20.9-31.2)	23.5 (19.0-29.9)	**.013**
LV ejection fraction, core lab (%)	54.0 (50.0-57.4)	53.0 (49.8-57.7)	54.2 (50.0-57.1)	54.7 (50.6-57.1)	.46
Mitral E velocity (cm/s)	85.5 (69.0-108.0)	93.5 (72.0-112.0)	83.0 (69.0-101.0)	83.0 (66.0-108.0)	**.008**

Mitral A velocity (cm/s)	76.0 (56.0-95.0)	71.0 (54.0-95.0)	72.0 (53.0-89.0)	83.0 (65.0-99.0)	**.002**
E/A ratio	1.1 (0.8-1.6)	1.2 (0.8-1.7)	1.1 (0.8-1.7)	1.0 (0.8-1.3)	**.004**
Septal e' velocity (cm/s)	6.0 (5.0-7.0)	6.0 (5.0-8.0)	6.0 (5.0-7.0)	6.0 (5.0-7.0)	.42
Lateral e' velocity (cm/s)	8.0 (6.0-10.0)	8.0 (6.0-10.0)	8.0 (7.0-10.0)	8.0 (6.0-10.0)	.34
Average E/e' ratio	12.4 (9.4-16.9)	13.1 (9.4-18.3)	11.9 (9.3-15.5)	12.5 (9.6-16.9)	.13
LA maximal volume, biplane (ml)	62.5 (49.0-82.8)	63.0 (51.7-84.9)	62.8 (48.5-84.8)	60.9 (47.4-79.8)	.17
RV end-diastolic diameter (cm)	3.7 (3.4-4.1)	3.8 (3.5-4.3)	3.8 (3.5-4.1)	3.6 (3.3-4.0)	**.016**
RV s' velocity (cm/s)	11.6 ± 3.1	11.1 ± 3.0	11.9 ± 3.1	11.8 ± 3.1	0.065
Tricuspid annular plane systolic excursion (cm)	2.0 (1.8-2.3)	2.0 (1.7-2.2)	2.0 (1.8-2.3)	2.0 (1.8-2.3)	.49
Right atrial volume (ml)	49.0 (37.0-66.0)	52.0 (41.0-72.0)	49.0 (35.0-65.0)	46.8 (35.0-63.0)	**.038**
Peak TR velocity (m/s)	2.6 (2.4-2.9)	2.7 (2.4-3.0)	2.6 (2.3-2.8)	2.6 (2.4-2.9)	.094
Estimated PA systolic pressure (mmHg)	31.3 (26.0-39.0)	33.2 (27.4-41.2)	30.9 (24.9-36.7)	31.0 (26.0-37.6)	.16
Estimated RA pressure (mmHg)	3.0 (3.0-3.0)	3.0 (3.0-3.0)	3.0 (3.0-3.0)	3.0 (3.0-3.0)	.63

Estimated stroke volume (ml)	67.8 (56.0-81.6)	66.1 (56.0-82.2)	68.7 (54.4-79.7)	65.9 (55.7-82.3)	.98
Estimated cardiac output (L/min)	4.4 (3.6-5.4)	4.3 (3.7-5.4)	4.4 (3.5-5.3)	4.8 (3.7-5.5)	.44
Degree of mitral regurgitation					.15
None	35 (7.4%)	9 (5.8%)	7 (4.2%)	19 (12.4%)	
Trace	198 (41.8%)	67 (43.2%)	71 (42.8%)	60 (39.2%)	
Mild	143 (30.2%)	46 (29.7%)	54 (32.5%)	43 (28.1%)	
Mild-to-moderate	54 (11.4%)	14 (9.0%)	23 (13.9%)	17 (11.1%)	
Moderate	34 (7.2%)	15 (9.7%)	9 (5.4%)	10 (6.5%)	
Moderate-to-severe	7 (1.5%)	2 (1.3%)	1 (0.6%)	4 (2.6%)	
Severe	3 (0.6%)	2 (1.3%)	1 (0.6%)	0 (0.0%)	
Degree of tricuspid regurgitation					.088
None	16 (3.4%)	4 (2.5%)	5 (3.1%)	7 (4.6%)	
Trace	165 (34.9%)	52 (32.3%)	54 (33.5%)	59 (39.1%)	
Mild	180 (38.1%)	53 (32.9%)	70 (43.5%)	57 (37.7%)	
Mild-to-moderate	52 (11.0%)	23 (14.3%)	19 (11.8%)	10 (6.6%)	
Moderate	50 (10.6%)	23 (14.3%)	11 (6.8%)	16 (10.6%)	
Moderate-to-severe	6 (1.3%)	4 (2.5%)	2 (1.2%)	0 (0.0%)	
Severe	4 (0.8%)	2 (1.2%)	0 (0.0%)	2 (1.3%)	
LV longitudinal strain (-%)	17.8 ± 3.5	17.9 ± 3.4	17.8 ± 3.7	17.7 ± 3.5	0.85

RV free wall strain (-%)	22.3 ± 5.7	22.6 ± 5.9	22.7 ± 5.5	21.8 ± 5.7	0.24
LA reservoir strain (%)	20.5 (14.2-27.1)	19.7 (14.2-26.1)	21.0 (13.9-28.1)	20.6 (14.3-26.7)	.59
RA reservoir strain (%)	24.0 (17.8-31.4)	23.5 (16.2-30.2)	24.9 (18.6-32.9)	23.8 (18.5-30.8)	.10
EAT thickness (mm)	5.5 (4.3-7.3)	3.9 (3.2-4.3)	5.5 (5.0-6.0)	8.2 (7.3-9.7)	**<.001**

Continuous data are presented as mean±SD for normally distributed variables and median (Q1, Q3) for non-normally distributed variables and n (%) for categorical measures. The Shapiro-Wilk test was used to determine normality of each variable. E/A, early to late mitral inflow velocities; e′, mitral annular early diastolic velocity; EAT, epicardial adipose tissue; LA, left atrial; LV, left ventricular; PA, pulmonary artery; RA, right atrial; RV, right ventricular; TAPSE, tricuspid annular plane systolic excursion; TR, tricuspid regurgitation

Supplemental Table 3: Hemodynamic parameters at 20W exercise

	Total N=566	1st tertile (2 – 5mm) N=187	2nd tertile (5 – 7mm) N=187	3rd tertile (7 – 17mm) N=192	p-value
Right atrial pressure (mmHg)	17.0 (13.0-21.0)	16.0 (12.0-20.0)	17.0 (13.0-21.0)	17.0 (14.0-21.0)	.20
PA systolic pressure (mmHg)	64.0 (54.0-75.0)	62.0 (54.0-73.0)	64.5 (55.0-76.0)	64.0 (54.0-75.0)	.72
PA diastolic pressure (mmHg)	30.0 (27.0-36.0)	30.0 (26.0-35.0)	30.0 (25.0-35.0)	32.0 (27.0-39.0)	**.038**
Mean PA pressure (mmHg)	41.7 (36.0-48.7)	40.7 (36.5-47.0)	41.8 (35.3-48.7)	43.3 (36.7-50.0)	.29
PCWP (mmHg)	32.0 (27.0-37.0)	31.0 (26.0-37.0)	32.0 (27.0-37.0)	32.0 (28.0-39.0)	.12
PCWP-right atrial pressure gradient (mmHg)	15.0 (11.0-19.0)	15.0 (11.0-19.0)	14.0 (11.0-19.0)	16.0 (12.0-20.0)	.15
RAP/PCWP ratio	0.5 (0.4-0.6)	0.5 (0.4-0.6)	0.5 (0.4-0.6)	0.5 (0.4-0.6)	.49
Transpulmonary gradient (mmHg)	9.7 (7.0-13.7)	9.7 (7.3-13.3)	9.7 (6.3-14.3)	9.7 (7.0-14.0)	.98

Continuous data are presented as mean±SD for normally distributed variables and median (Q1, Q3) for non-normally distributed variables. The Shapiro-Wilk test was used to determine normality of each variable. CO, cardiac output; PA, pulmonary artery; PCWP, pulmonary capillary wedge pressure; RAP, right atrial pressure WU, Wood units.

Supplemental Table 4: Change in invasive hemodynamic parameters from rest to peak exercise.

	Total N=566	1st tertile (2 – 5mm) N=187	2nd tertile (5 – 7mm) N=187	3rd tertile (7 – 17mm) N=192	p-value
Delta RA pressure (mmHg)	28.6 ± 12.0	29.2 ± 12.4	28.7 ± 11.8	28.0 ± 11.7	0.67
Delta PA systolic pressure (mmHg)	15.0 ± 6.9	14.8 ± 7.2	14.8 ± 7.0	15.4 ± 6.4	0.68
Delta PA diastolic pressure (mmHg)	19.5 ± 7.6	19.6 ± 7.9	19.5 ± 7.7	19.6 ± 7.3	0.98
Delta Mean PA pressure (mmHg)	19.3 (14.3-24.3)	19.0 (15.0-24.7)	19.7 (14.3-24.0)	19.3 (14.7-24.3)	.99
Delta PCWP (mmHg)	16.0 (12.0-21.0)	17.0 (11.0-21.0)	16.0 (12.0-20.0)	16.0 (12.0-22.0)	.38
Delta PCWP–right atrial pressure gradient (mmHg)	7.0 (3.0-12.0)	6.0 (2.0-11.0)	7.0 (2.0-11.0)	9.0 (3.0-12.0)	**.022**
Delta RAP/PCWP ratio (mmHg)	0.0 (-0.1-0.1)	0.0 (-0.1-0.1)	0.0 (-0.1-0.1)	-0.0 (-0.1-0.1)	.058
Delta transpulmonary gradient (mmHg)	2.7 (-0.3-6.3)	2.7 (-0.3-6.7)	3.0 (-0.0-6.3)	1.8 (-0.7-6.0)	.30
Delta stroke volume (ml)	-21.0 (-29.9--12.9)	-22.0 (-30.6--13.9)	-20.4 (-29.2--12.7)	-20.3 (-28.7--12.5)	.73
Delta stroke volume index (ml/m2)	-10.4 (-14.4--6.2)	-11.0 (-14.9--6.9)	-9.8 (-14.0--6.2)	-9.8 (-14.5--6.0)	.51

Delta cardiac output (L/min Baim adjudicated)	2.7 (1.6-4.3)	2.7 (1.6-4.3)	3.0 (1.6-4.3)	2.7 (1.6-4.0)	.70
Delta cardiac index (L/min/m2)	1.4 (0.8-2.0)	1.4 (0.8-2.0)	1.4 (0.8-2.1)	1.3 (0.8-2.0)	.89
Delta systemic vascular resistance (WU)	-5.1 (-7.6--2.7)	-5.3 (-8.4--2.6)	-5.1 (-7.3--2.6)	-5.0 (-7.1--3.0)	.77
Delta PVR (WU Baim adjudicated)	-0.2 (-0.6-0.3)	-0.2 (-0.5-0.3)	-0.1 (-0.6-0.4)	-0.3 (-0.7-0.2)	.14
Delta PA pulse pressure/stroke volume ratio (mmHg/ml)	0.3 (0.2-0.5)	0.4 (0.2-0.6)	0.3 (0.2-0.5)	0.3 (0.2-0.5)	.064

Continuous data are presented as mean±SD for normally distributed variables and median (Q1, Q3) for non-normally distributed variables. The Shapiro-Wilk test was used to determine normality of each variable. CO, cardiac output; PA, pulmonary artery; PCWP, pulmonary capillary wedge pressure; PVR, pulmonary vascular resistance; RAP, right atrial pressure WU, Wood units.
